# Antibacterial Calcium Phosphate Coatings for Biomedical Applications Fabricated via Micro-Arc Oxidation

**DOI:** 10.3390/biomimetics8050444

**Published:** 2023-09-21

**Authors:** Anna I. Kozelskaya, Ksenia N. Verzunova, Igor O. Akimchenko, Johannes Frueh, Vsevolod I. Petrov, Galina B. Slepchenko, Olga V. Bakina, Marat I. Lerner, Leonid K. Brizhan, Denis V. Davydov, Artur A. Kerimov, Elena G. Cherempey, Sergey E. Krylov, Sven Rutkowski, Sergei I. Tverdokhlebov

**Affiliations:** 1 Weinberg Research Center, School of Nuclear Science &Engineering, Tomsk Polytechnic University, 30, Lenin Avenue, 634050 Tomsk, Russia; shumskaya_k@mail.ru (K.N.V.); akimchenko.igor@gmail.com (I.O.A.); johannes.frueh@alumni.ethz.ch (J.F.); microlab@tpu.ru (G.B.S.); 2 Tomsk Scientific Center of the Siberian Branch of the Russian Academy of Sciences, 10/4, Akademicheskii Prospekt, 634055 Tomsk, Russia; seva-ne@mail.ru; 3 Institute of Strength Physics and Materials Science of the Siberian Branch of the Russian Academy of Sciences, 2/4, Akademicheskii Prospekt, 634055 Tomsk, Russia; ovbakina@ispms.ru (O.V.B.); lerner@ispms.ru (M.I.L.); 4 Federal State Budgetary Institution «Main Military Clinical Hospital Named after Academician N.N. Burdenko» of the Ministry of defense of the Russian Federation», 3 Gospitalnaya Square, 105299 Moscow, Russia; brizhan.leonid@mail.ru (L.K.B.); dvdavydov@yandex.ru (D.V.D.); kerartur@ya.ru (A.A.K.); 5 UMH LLC, 48 Govorova Street, Apt. 191, 634050 Tomsk, Russia; cherempey@mail.ru; 6 BITECA LLC, 9 Zapadnaya Street, Building 10, 143002 Odintsovo, Russia; info@biteca.ru

**Keywords:** bioactive coatings, antibacterial coatings, calcium phosphate coatings, micro-arc oxidation, zinc oxide, biocompatibility, antibacterial properties, plasma electrolytic oxidation, Gram-positive bacteria, Gram-negative bacteria

## Abstract

A promising method for improving the functional properties of calcium-phosphate coatings is the incorporation of various antibacterial additives into their structure. The microbial contamination of a superficial wound is inevitable, even if the rules of asepsis and antisepsis are optimally applied. One of the main problems is that bacteria often become resistant to antibiotics over time. However, this does not apply to certain elements, chemical compounds and drugs with antimicrobial properties. In this study, the fabrication and properties of zinc-containing calcium-phosphate coatings that were formed via micro-arc oxidation from three different electrolyte solutions are investigated. The first electrolyte is based on calcium oxide, the second on hydroxyapatite and the third on calcium acetate. By adding zinc oxide to the three electrolyte solutions, antibacterial properties of the coatings are achieved. Although the same amount of zinc oxide has been added to each electrolyte solution, the zinc concentration in the coatings obtained vary greatly. Furthermore, this study investigates the morphology, structure and chemical composition of the coatings. The antibacterial properties of the zinc-containing coatings were tested toward three strains of bacteria—Staphylococcus aureus, methicillin-resistant Staphylococcus aureus and Pseudomonas aeruginosa. Coatings of calcium acetate and zinc oxide contained the highest amount of zinc and displayed the highest zinc release. Moreover, coatings containing hydroxyapatite and zinc oxide show the highest antibacterial activity toward *Pseudomonas aeruginosa*, and coatings containing calcium acetate and zinc oxide show the highest antibacterial activities toward *Staphylococcus aureus* and methicillin-resistant *Staphylococcus aureus*.

## 1. Introduction

Biomedical implants have paved the way for medicine and changed the lives of many people [1]. Numerous surgical procedures are performed annually to treat fractures and bone deformities [2]. Modern regenerative medicine is currently focused on the regeneration of pathologically altered tissue [3]. This is achieved by replacing a bone defect with an implant placed in the affected area [4]. Such treatments shorten recovery times and improve the quality of life of patients [5]. Titanium-based alloys are currently considered to be the most promising implant materials because of their high corrosion resistance, high specific strength, low density and excellent mechanical properties [1].

To improve the osseointegration of metal implants, calcium phosphate coatings are commonly applied to their surface [6]. From a clinical point of view, early ingrowth of the implant with the surrounding living bone tissue promotes the onset of patient activity shortly after operation, resulting in promising long-term healing outcomes [7].

Osteosynthesis surgical procedures encounter problems related to suboptimal osseointegration of implants into the surrounding bone tissue and the risk of antibiotic-resistant bacteria entering the patient’s wound tissue [8]. According to the literature, microbial contamination of the surgical wound is inevitable even if the rules of asepsis and antisepsis are optimally followed [9]. In recent years, to deal with antibiotic-resistant bacteria, various metal ions have been incorporated into the structure of calcium phosphate coatings, which exhibit prolonged drug release, low cytotoxicity, great selectivity, and heat resistance, unlike organic antibiotics [10,11,12].

There are multiple methods to improve the osseointegration of metal implants into the patient’s bone tissue while maintaining antibacterial properties; these include mechanical surface treatment methods and coatings [13]. These methods include sol-gel process [14], physical vapor deposition [15], chemical vapor deposition [16], plasma spraying [17], laser cladding [18], anodizing [19], pulsed laser deposition [20], matrix-assisted pulsed laser evaporation [21] and plasma electrolytic oxidation (PEO), also known as microarc oxidation (MAO) [22]. It has been reported by Geng et al. that zinc-doped calcium-phosphate (CaP)-based coatings fabricated via magnetron sputtering on Ti–6Al–4V and Ti–6Al–7Nb alloys reduced the growth of *Staphylococcus aureus* and leukemic Jurkat T cells [23]. Antibacterial efficacy toward *Staphylococcus aureus* was also demonstrated by Zn- or Cu-containing CaP-based coatings formed via MAO on titanium and the alloy, Ti-40Nb [24]. Hydrothermally deposited CaP coatings doped with silver (Ag) and strontium (Sr) have also exhibited good antibacterial activities toward both *Escherichia coli* and *Staphylococcus aureus* [25].

Coatings fabricated via MAO have attracted much attention [1,26,27,28]. The most significant advantage of MAO coatings for use in biomaterials is the presence of pores on the coating surface and the ability to control the pore size by refining the process parameters [29,30]. This is because the composition of a MAO coating can also be changed by tailoring the electrolyte solution. The addition of additives to an electrolyte solution can influence the MAO process to significantly improve surface properties, such as wear and corrosion resistance, antibacterial properties, and biocompatibility [31]. In addition, the porous surface of MAO coatings is suitable for the release of antibiotics to treat infections after surgery [32]. Good substrate adhesion, high hardness, the ability to uniformly coat metal implants with complex geometries, simple equipment, and low cost are key advantages of this method [29,31].

From the analysis of literature, the most commonly used antibacterial additives are silver (Ag^+^), copper (Cu^2+^), and zinc (Zn^2+^) ions [33]. Reference [34] shows that these metal ions are arranged as follows with respect to their cytotoxic impact: Ag^+^ > Cu^2+^ > Zn^2+^. Moreover, zinc is a component of enzymes in living organisms or acts as its activator, and is also important for the immune system and has antibacterial and anti-inflammatory properties [35]. Zinc ions (Zn^2+^) exhibit antimicrobial activity towards various strains of bacteria, viruses, and fungi [12,36,37]. In addition to the activity of water-soluble zinc compounds (e.g., zinc acetate), ZnO combines two other mechanisms of antimicrobial activity to complement its effectiveness: the generation of reactive oxygen species (ROS) and direct contact with cell walls [38]. The aim of the present study is to evaluate the antimicrobial activity of zinc-containing calcium phosphate coatings formed in three different electrolyte solutions via micro-arc oxidation and tests towards three different strains of bacteria.

## 2. Materials and Methods

**Figure 1:**
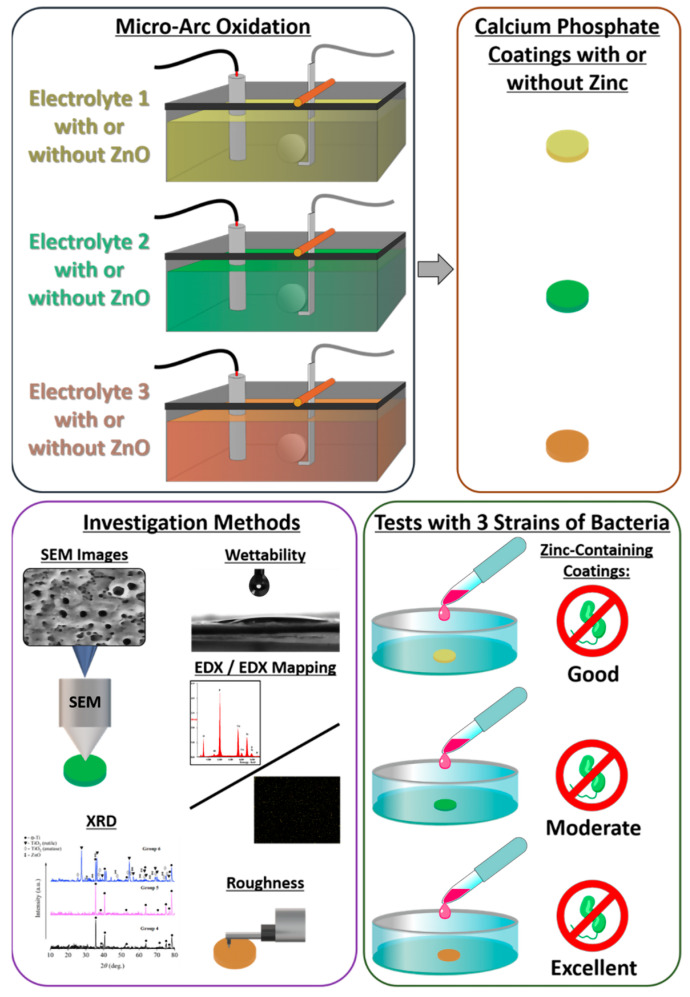
Schematic overview of the surface modification of titanium sample discs via micro-arc oxidation (MAO) with three different electrolyte solutions (one time without zinc oxide (ZnO) and a second time with ZnO added to the three different electrolyte solutions), as well as schematic representation of the utilized investigation methods: scanning electron microscopy (SEM), wettability measurement, energy-dispersive X-ray (EDX) analysis and mapping, X-ray diffraction (XRD), and roughness measurement, as well as the antibacterial assays of the zinc-containing coatings towards three bacteria strains, *Pseudomonas aeruginosa*, *Staphylococcus aureus*, and methicillin-resistant *Staphylococcus aureus*.

An overview of the samples and their surface modification, as well as the applied investigation methods are shown schematically in Figure 1.

*Fabrication of the Coatings:* Titanium discs made of Ti6Al4V with a diameter of 10 mm and a thickness of 1 mm were used as substrates for the micro-arc oxidation (MAO) coatings. Before applying the coatings, the surface of the samples was chemically etched in an aqueous solution of nitric and hydrofluoric acids in the volume ratio, HNO_3_:HF:H_2_O = 1:2.5:2.5, at a temperature of 15–20 °C for 10–15 s, followed by neutralization in 1% aqueous sodium hydroxide solution and rinsing several times with distilled water. Formation of calcium-phosphate coatings was carried out on a MAO setup developed at the Laboratory of Plasma Hybrid Systems of Tomsk Polytechnical University and Tomsk Scientific Center of SB RAS in cooperation with OOO Microsplav (Tomsk, Russia). Coating of samples was carried out in three different electrolyte solutions:

1. The first type of electrolyte solution contained 27 g/L (0.48 mol/L) calcium oxide (CaO, reagent grade, Component-Reaktiv, LLC, Moscow, Russia), 10 g/L (0.01 mol/L) hydroxyapatite (Ca_10_(PO_4_)_6_(OH)_2_, reagent grade, Fluidinova, Maia, Portugal) and 70 mL/L (0.07 mol/L) orthophosphoric acid (H_3_PO_4_, reagent grade, Component-Reaktiv, LLC, Moscow, Russia). Samples with coatings formed in this electrolyte solution will be referred to as “Electrolyte 1” in the following.
2. The second type of electrolyte solution contained 40 g/L (0.04 mol/L) hydroxyapatite (Ca_10_(PO_4_)_6_(OH)_2_, chemically pure, BITECA LLC, Odintsovo, Russia) and 70 mL/L (0.07 mol/L) orthophosphoric acid (H_3_PO_4_, reagent grade, Component-Reaktiv, LLC, Moscow, Russia). Samples with coatings formed in this electrolyte solution will be referred to as “Electrolyte 2” in the following.
3. A solution of 36.8 g/L (0.21 mol/L) calcium acetate monohydrate (Ca(CH_3_CO_2_)_2_ · H_2_O, reagent grade, Component-Reaktiv, LLC, Moscow, Russia), 15.6 g/L (0.11 mol/L) (sodium phosphate monohydrate (NaH_2_PO_4_∙H_2_O, reagent grade, VEKTON JSC, Saint Petersburg, Russia) and 1.5 mL/L (0.002 mol/L) orthophosphoric acid (H_3_PO_4_, reagent grade, Component-Reaktiv, LLC, Moscow, Russia) was used as the third electrolyte solution. Samples with coatings formed in this electrolyte solution will be referred to as “Electrolyte 3” in the following.

Additionally, samples marked “+ZnO” refer to the coatings which were formed in the same three electrolyte solutions mentioned above, but to each of which, 5 g/L (0.12 mol/L) zinc oxide (chemically pure, Alfahim plus LLC, Saint Petersburg, Russia) was added. The average particle size of the zinc oxide powder used is (3.8 ± 0.2) μm and was determined via laser particle size analyzer (SALD-7101, Shimadzu, Kyoto, Japan). All electrolyte solutions were prepared using distilled water, which was produced using an electric water distiller (DE-10M, ZAVOD EMO LLC, Saint Petersburg, Russia).

MAO process parameters for the formation of coatings for the samples, Electrolyte 1, Electrolyte 2, Electrolyte 1 + ZnO, and Electrolyte 2 + ZnO were as follows:—voltage: 300 V, —voltage rise rate: 3 V/s, —pulse repetition frequency: 200 Hz, —pulse duration: 100 μs, —coating formation time: 15 min. Process parameters for the formation of coating formation for the samples, Electrolyte 3 and Electrolyte 3 + ZnO, were as follows:—voltage: 500 V, —voltage rise rate: 3 V/s, —pulse repetition frequency: 500 Hz, —pulse duration: 400 μs, —coating formation time: 3 min. The photographs of the samples surface-modified by micro-arc oxidation are shown in the supporting information (SI) manuscript Figure S1.

*Characterization of the Coatings*: Coating thicknesses were investigated via eddy-current method, using a portable thickness meter (Constanta K5, OOO Constanta, Saint Petersburg, Russia). Roughness measurements on the coating samples were carried out using a profilometer (Talysurf 5-120, Tailor-Hobson, Leicester, England). Numerical evaluation of the surface roughness was evaluated via the arithmetic mean deviation (*R_a_*) and the height of profile irregularities at ten points of the surface profile (*R_z_*). Wettability of the sample surfaces have been measured on a drop shape analyzer (Easy Drop DSA 20, Krüss, Hamburg, Germany) using the Drop Shape Analysis software (version 1.92.1.1, Krüss, Hamburg, Germany). For this purpose, the liquids distilled water (Solopharm, Saint Petersburg, Russian Federation), diiodomethane (99%, Acros Organics, Geel, Belgium) and glycerol (Ekos-1, Moscow, Russian Federation) were used. Five droplets with a volume of 3.0 µL of each liquid were placed on the sample surfaces to obtain the water contact angles (WCA) and diiodomethane contact angles (DCA). The obtained contact angles were used to calculate surface energies according to the Owens-Wendt-Rabel-Kaelble (OWRK) method. Morphology and elemental composition of the coatings were investigated via scanning electron microscopy (SEM; Quanta 200 3D, FEI Company, Hillsboro, OR, USA) energy dispersive X-ray spectroscopy (EDX; EDAX ECON-4, EDAX, Mahwah, NJ, USA). EDX measurements were performed under high vacuum with an accelerating voltage of 20 kV. Phase compositions of the coatings were studied via X-ray diffraction (XRD) analysis using a Shimadzu XRD 6000 X-ray diffractometer (Shimadzu, Kyoto, Japan) on CuKα radiation (1.5406 Å) in standard Bragg–Brentano geometry. The following parameters were applied for the XRD measurements: X-ray tube voltage—40 kV, X-ray tube current—30 mA, scanning angle range—10–80°, scanning step size—0.0200 and a signal collection time of—1 s. Calculations of the lattice parameters and phase relations were performed using a full-profile analysis software (Powder Cell 2.4, Federal Institute for Materials Research and Testing, Berlin, Germany). Zinc release was investigated by means of stripping voltammetry. This measurement is described in details in the Supplementary Materials and methods chapter of the SI manuscript.

*Antibacterial Properties:* Antimicrobial activities of the samples were assessed via the droplet contamination method, according to ISO 22196:2011, using the following three strains of bacteria: *Staphylococcus aureus* ATCC 6538-P (*S. aureus*), *Pseudomonas aeruginosa* ATCC 27853 (*P. aeruginosa*), and methicillin-resistant *Staphylococcus aureus* ATCC 43300 (MRSA). Three samples of each sample type and three control samples were used. The samples were placed in a sterile 24-well plate. A volume of 64 μL (1 µL per 1 mm^2^ sample area) of the bacterial suspension with a concentration 10^5^ CFU/mL was applied to the samples surface and spread evenly on the surface using a sterile swab. As a control, three empty wells plates were inoculated with the 64 μL of the test culture suspension on the surface of titanium samples without a coating (to form control samples). The exposure time was 6 h. A further increase in the exposure time led to a decrease in the viability of bacteria in the control sample. After 6 h of exposure, samples were placed in sterile containers containing 10 mL of neutralizing agent (0.9% sodium chloride solution, Grotex, Moscow, Russia) and shaken for 10 min using an orbital shaker (PSU-20i, SIA Biosan, Riga, Latvia) to remove adhered bacteria. From each supernatant, 100 µL were aliquoted onto Mueller–Hinton agar (HiMedia Laboratories, Mumbai, Maharashtra, India) plates. Then, the supernatant was diluted by factors of 10, 100 and 1000 and also spread onto Mueller–Hinton agar plates. The plates were subsequently incubated at 37 ± 1 °C for 24 h. The colony plate count method was used for the enumeration of CFUs after incubation. Antibacterial activities were evaluated by calculating the percent reduction in microbial contamination of test samples compared to the control samples according to the following equation [39]:

(1) $$Antibacterial\;activity\;\left(\%\right)=100\cdot \left(\left(A-B\right)/A\right)$$

where *A* is the number of viable bacteria in the control samples and *B* is the number of viable bacteria in the experimental samples.

## 3. Results and Discussion

**Figure 2:**
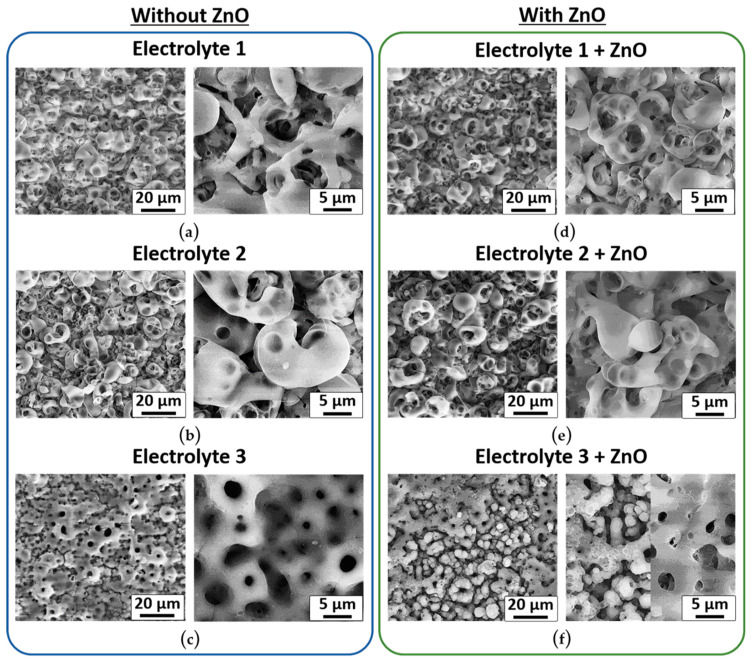
SEM micrographs of the microscopic appearance of three different calcium-phosphate coatings on top of a titanium substrate formed by micro-arc oxidation (MAO). (**a**) Coating formed using Electrolyte 1; (**b**) coating formed using Electrolyte 2; (**c**) coating formed using Electrolyte 3. (**d**–**f**) coatings were formed using the Electrolytes 1–3, with the addition of zinc oxide (ZnO). For more details of the electrolyte compositions, see chapter 2 (Materials and Methods).

**Figure 3:**
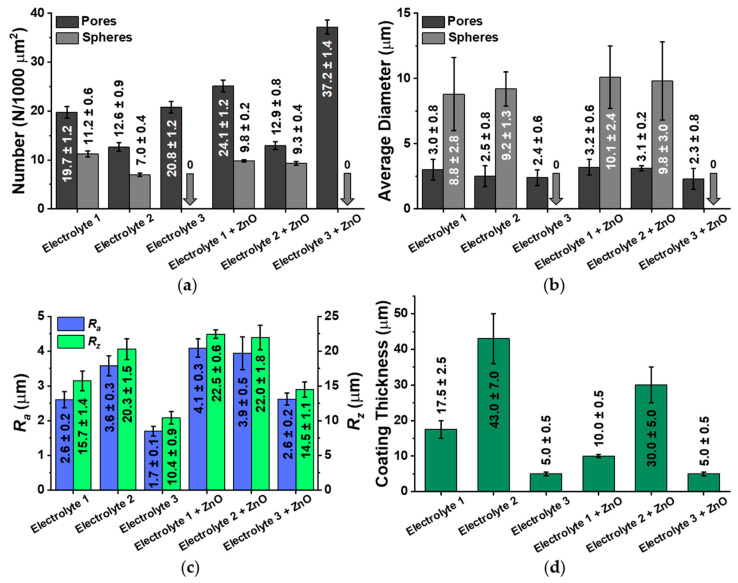
(**a**) Amount of pores and spheres for each sample. (**b**) Average pore and sphere size of the investigated sample types. (**c**) Surface roughness shown as *R_a_* (arithmetic mean roughness) and *R_z_* (average height difference) investigated via contact profilometry. (**d**) Obtained coating thicknesses.

*Surface Morphology:* The surface morphology of the calcium phosphate coatings of the samples Electrolyte 1, Electrolyte 2, Electrolyte 1 + ZnO, and Electrolyte 2 + ZnO shows hollow spherulites with an average size of about 10 μm uniformly distributed on the surface of the samples (Figure 2a,b,d,e and Figure 3a,b). With the addition of zinc oxide, no changes on the surface morphology of these samples are observed. Surfaces of the samples Electrolyte 3 and Electrolyte 3 + ZnO differ from those observed from other sample groups and have a structure similar to a volcanic crater with a pore in the center, or the so-called “pancake-like” structure (Figure 2c,f). This structure has also been reported by other researchers [40,41,42,43,44]. The average pore diameter of Electrolyte 3 and Electrolyte 3 + ZnO coatings is about 3 µm (Figure 2c,f). It should be noted that the surface of Electrolyte 3 + ZnO coatings is heterogeneous. There are areas with coarse surface topography, reminiscent of volcanic craters, as well as areas with particle agglomerates adhering as a result of a thermochemical reaction (a less coarse relief). The agglomerated particles are not containing zinc and have a composition similar to the rest of the coating areas. These areas are described in more detail below in the Chemical Composition of the Coatings section, and their elemental mapping can be seen in SI Figure S2.

The numerical characteristics of the surface relief of the samples are shown in Figure 3a–c as number of pores and spheres, diameter of pores and spheres, as well as roughness types *R_a_* (arithmetic mean roughness) and *R_z_* (average height difference). Thicknesses of calcium-phosphate coatings formed with Electrolyte 1, Electrolyte 2, Electrolyte 1 + ZnO and Electrolyte 2 + ZnO ranged from 10 to 43 µm (Figure 3c). In contrast, the coating thicknesses for the Electrolyte 3 and Electrolyte 3 + ZnO samples are in the range of 5 µm (Figure 3c). These large variations in the thicknesses of the coatings formed in different electrolyte solutions are due to both the different chemical compositions of the electrolyte solutions and the applied MAO process modes under which the coatings were formed. It is known that the electrolyte composition also affects the surface morphology and properties of the formed coatings [31,45,46]. The MAO process parameters applied also have a strong influence on the performance of the coatings [47,48]. In this study, the electrolyte compositions and the MAO process modes used were chosen to generate a porous surface morphology of the coatings for better cell attachment.

It is interesting to note that the Electrolyte 1 and Electrolyte 2 coatings display a decrease in their coating thickness when ZnO is added, while this is not the case for the Electrolyte 3 coatings. This due to the fact that the formation of the Electrolyte 3 and Electrolyte 3 + ZnO coatings occurs within 3 min, in contrast to the other coatings, which are formed within 15 min. Therefore, the effect may not be noticeable with such a short coating time. The formation of Electrolyte 3 + ZnO coatings occurs at a higher voltage and a longer pulse duration time. In general, the stronger the current flows through the coated substrate, the thicker the formed coating becomes. On the other hand, the MAO process consists of a series of complex electrochemical reactions in which the particles from the electrolyte solution “adhere” on the substrate surface during the solidification of the molten substrate material also contribute to the thickening of the forming coating. In the case of Electrolyte 3 + ZnO samples, the particles located near the cooling substrate are likely to dissociate into smaller particles, making their contribution to the resulting coating thickness smaller compared to the formation of coatings formed with other electrolyte solutions and under “gentler” MAO process parameters.

The decrease in the thickness of the Electrolyte 1 + ZnO and Electrolyte 2 + ZnO coatings with the addition of zinc may also be due to the fact that a temperature of 1975 °C is required for the melting of zinc oxide, while the melting temperature of titanium oxide is 1843 °C, which is 132 °C lower. In general, it is quite difficult to clearly determine which main factor influences the thickness of the coatings. As this discussion has pointed out, it should rather be assumed that there is an interplay of several different factors.

It can be seen from Figure 3a that the incorporation of Zn^2+^ in the coatings correlates with an increase in pore number, but not in spheres. An increase in the zinc concentration leads to a higher number of breakdowns, which results in the formation of pores on the coating surface. As the number of pores increases, the roughness values of the coatings also increases (Figure 3d). This correlation is observed for all MAO samples in this study, except for the Electrolyte 2 samples. The number of pores on the surface of the Electrolyte 2 + ZnO coatings does not change compared to the Electrolyte 2 samples and consequently the roughness value does not change. It should also be noted that the addition of ZnO to the electrolyte solutions does not lead to an increase in the average diameter of the formed pores and spheres of the investigated coatings (Figure 3b).

*Chemical Composition of the Coatings:* Investigation of the elemental composition of the coatings fabricated via energy dispersive X-ray spectroscopy (EDX) shows that the coatings consist of an elemental composition present in the electrolyte solutions (calcium—Ca, phosphorus—P, oxygen—O, zinc—Zn) and in the substrate (titanium—Ti, vanadium—V, aluminum—Al) (Table 1). It should be noted that the highest value of Ca/P ratio is observed for the sample Electrolyte 3. A possible reason for the low Ca/P ratio of the coatings formed in Electrolyte 1 and Electrolyte 2 is described below.

The process of coating formation via micro-arc oxidation (MAO) is affected by a strong electric field between the anode (sample) and the cathode (bath walls containing the respective electrolyte solution, see Figure 1). As reported in the literature, the electric field strength in the plasma layer near the sample can reach 10^4^–10^8^ V/m during the MAO process [49,50,51]. Under the action of an electric field of this strength, electrolyte anions will be incorporated into the coating being formed. The solutions of Electrolyte 1 and Electrolyte 2 may contain the following dissolved ions: H_2_PO_4_^−^, H^+^, Ca^2+^, H_2_O, HPO_4_^2^, CaH_2_PO_4_^+^, PO_4_^3−^, OH^−^, Ca_10_(PO_4_)_6_OH^+^, Ca_10−x_(PO_4_)_6_^2(1−x)+^, etc. Negatively charged ions are attracted to the positively charged sample. Neutral particles and positively charged ions apparently penetrate the coating due to other reactions, including “welding” to the surface [52]. Therefore, the phosphorus content predominates over the calcium content in deposited coatings. In the case of Electrolyte 3, the electrolyte solution appears to be more dominated by calcium-containing ions and neutral compounds. As a result, mainly coatings with a high Ca/P ratio are formed (Table 1).

The addition of ZnO powder to the Electrolyte 3 solution leads to the formation of zinc-containing coatings with a lower Ca/P ratio than without zinc (Table 1). As it can also be seen in Table 1, zinc is much more present in the coatings formed with Electrolyte 3 + ZnO. Consequently, the (Zn + Ca)/P ratio is also high for all zinc-containing coatings. This effect can be explained by the different micro-arc oxidation parameters that were applied. As mentioned in chapter 2, the applied voltage, pulse repetition frequency and pulse duration time for the formation of Electrolyte 3 + ZnO coatings were much higher than in the case of the Electrolyte 1 + ZnO and Electrolyte 2 + ZnO coatings. In this case, the ZnO in the Electrolyte 3 solution was more ionized and more Zn^2+^ ions can be formed near the sample surface, which can then be incorporated into the forming coating (plasma chemical reaction).

Elemental mapping (EDX mapping; SI Figure S2) shows a uniform distribution of the elements oxygen (O), phosphorus (P), calcium (Ca), titanium (Ti), and zinc (Zn) for the zinc-containing coatings. A slight heterogeneity in the distribution of Ti in the Electrolyte 3 + ZnO coatings is associated with the characteristics of the growth of MAO coatings. In the SEM micrographs of Electrolyte 3 + ZnO coatings and in the titanium EDX mapping micrograph for these coatings (SI Figure S2), it can be seen that where the amount of Ti on the surface is greater, there are areas of coarser surface topography resembling volcanic craters (or a “pancake-like” structure) with a micropore in the center. On the contrary, in places with a lower Ti concentration, regions with a less coarse relief are observed. Moreover, in these places all detected elements are evenly distributed. All this indicates that there were breakdown sites of the dielectric layer in the areas with the most pronounced relief. Thus, with sufficient voltage, there is a breakdown of the formed dielectric oxide layer. Microdischarge channels are formed at the breakdown site, where temperature and pressure rise to tens of thousands of Kelvin (K) and thousands of megapascals (MPa), respectively [1,50]. As a result, the molten substrate material is removed from the microdischarge channels along with the dopants and reaches the coating surface in contact with the cooled electrolyte, resulting in rapid solidification and local thickening of the coating. Another breakdown of the dielectric oxide layer occurs at another location with a thinner coating, where the resistance to the voltage is lower and the voltage is therefore sufficient for a breakdown. For this reason, a dielectric breakdown occurs at different locations on the sample surface. Non-uniform coating growth on the surface of complex-shaped 3D-printed samples during the formation of a MAO coating has already been reported [53]. As the duration of the MAO process increases, the coating thickness usually becomes uniform over the entire sample surface.

**Figure 4:**
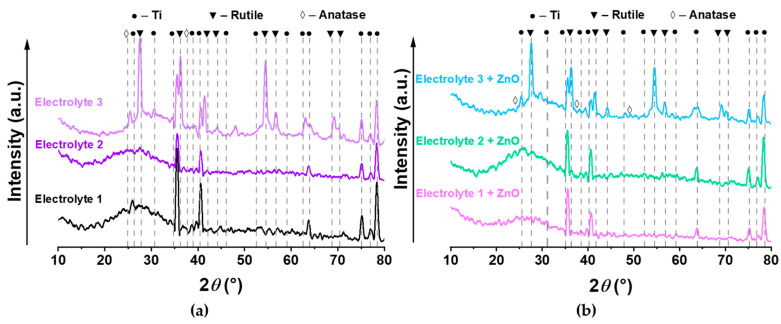
X-ray diffraction (XRD) patterns of the surface-modified samples prepared (**a**) without and (**b**) with the addition of zinc oxide (ZnO).

*Phase Composition of the Coatings:* The presence of both, long narrow peaks and a diffuse halo in the diffraction patterns of all investigated coatings indicates amorphous-crystalline structure. In particular, the diffraction patterns of all coatings contain peaks for crystalline titanium phases (Figure 4). The coatings formed with Electrolyte 3 and Electrolyte 3 + ZnO reveal rutile and anatase peaks of titanium dioxide (TiO_2_) formed from the titanium of the substrate and oxygen from the electrolyte solutions (Figure 4a,b). The crystalline ZnO phase is not present in the XRD spectra of the zinc-containing coatings (Figure 4b). A diffuse halo in the angular range 2*θ* = 15°–35° is observed in the diffraction patterns of all coatings, which indicates an amorphous calcium-phosphate structure [54,55,56,57,58]. In accordance with the area under the peaks of the crystalline phase and the diffuse halo, the percentage of the amorphous phase in the studied coatings varies from 16% for the coatings prepared with Electrolyte 1 + ZnO to 28% for the coatings fabricated with Electrolyte 2. In general, the coatings display the expected X-ray diffraction peaks known for this type of coating (Figure 4a) [53].

**Figure 5:**
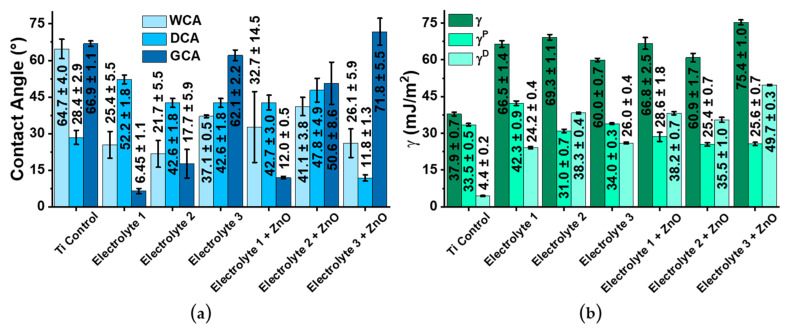
(**a**) Wettability of all investigated samples represented by water contact angles (WCA), diiodomethane contact angles (DCA) and glycerol contact angles (GCA). (**b**) Surface energy (γ) of the samples, which is also presented by the disperse component of the surface energy γ^D^ and the polar component γ^P^. Please note that the titanium samples are unmodified and serve as control samples.

*Wettability and Surface Energy of the Samples:* Comparing the contact angles of water and diiodomethane, a clear difference between the different coatings is evident (Figure 5 and SI Figure S3). Electrolyte 1 and Electrolyte 2 samples have better water wettability than Electrolyte 3 samples (Figure 5). The addition of zinc oxide to electrolyte 1 solution does not change the wettability of the coatings formed. In turn, the addition of zinc oxide to the Electrolyte 2 solution leads to the formation of coatings whose wettability is reduced (the water contact angle doubles). The wettability of coatings formed using the Electrolyte 3 solution and zinc oxide increases slightly compared to coatings without zinc. There is a general trend towards an increase in the wettability (water contact angles decrease) of the surface-modified samples compared to the unmodified titanium samples. The Electrolyte 1 and Electrolyte 1 + ZnO samples are characterized by an approximately equal surface energy γ. However, the addition of zinc oxide leads to the dominance of the disperse component γ^D^ of the surface energy γ. If zinc oxide is added to the Electrolyte 2 solution, the surface energy γ of the formed Electrolyte 2 + ZnO coatings decreases. In this case, the disperse component γ^D^ does not change. An increase in surface energy γ occurs when zinc oxide is added to the Electrolyte 3 solution. There is also a change from the polar component of the surface energy γ^P^ to the disperse component γ^D^.

**Figure 6:**
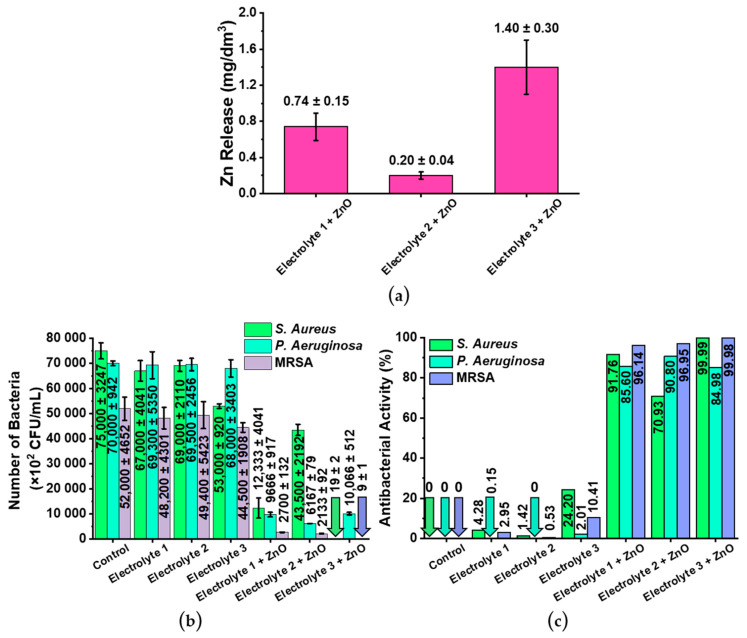
Investigation of the antibacterial properties of all investigated samples. (**a**) Zinc release from the zinc-containing samples after 6 h. (**b**) Number of the bacteria *S. aureus*, *P. aeruginosa*, and MRSA in colony-forming units per milliliter (CFU/mL). (**c**) Antibacterial activities of the investigated samples against the three different bacteria. Please note, uncoated titanium samples were used as control samples.

*Antibacterial Properties of the Coatings:* Comparing the zinc release from zinc-containing coatings, a clear dependence of the Zn^2+^ release on the type of electrolyte solution is observed (Figure 6a). This finding can be explained by the inherent zinc content between the coatings, as Table 1 indicates. Another conclusion that can be drawn from the results (Figure 6) is that zinc is an effective antibacterial agent against both Gram-positive (*S. aureus*, MRSA) and Gram-negative bacteria (*P. aeruginosa*). It should also be noted that the calcium phosphate coatings without the addition of zinc have a low antibacterial effect compared to the coatings with zinc (Figure 6b,c). This finding is in agreement with the existing literature [59] and confirms the approach used to obtain antibacterial anti-biofouling coatings. All samples showed the highest antibacterial activity towards MRSA bacteria (96.14–99.98% reduction). The antibacterial properties of the zinc-containing samples towards *Staphylococcus aureus* are directly related to the amount of zinc released, as shown in Figure 6b (columns for *S. aureus*). It is noted that despite the lowest zinc release in solution from the coatings, the antibacterial activity of the Electrolyte 2 + ZnO samples towards *Pseudomonas aeruginosa* is not significantly different from the Electrolyte 3 + ZnO samples with the highest zinc release. Among the reported antibacterial mechanisms of ZnO is the release of Zn^2+^ by ZnO-induced reactive oxygen species (ROS) [60]. In this reaction, ROS as well as Zn^2+^ ions attack the negatively charged bacterial cell wall and cause damages to the cell wall resulting in leakages from the cell and consequent mortality of the bacteria [61]. Da Silva et al. [62] demonstrated that higher ZnO concentrations are required for inhibition of Gram-negative bacteria than for Gram-positive strains. Gram-negative bacteria (including *Pseudomonas aeruginosa*) have a thin peptidoglycan layer between two membranes and a lipopolysaccharide layer [63], which is known to provide antimicrobial resistance [64]. This layer acts as a barrier preventing the intrusion of ROS and Zn^2+^ ions. Therefore, it is not possible to determine a correlation between antibacterial activity and ion release.

Comparing the results of this study with the biocompatibility requirements of implants, it can be seen that the cytotoxic Zn^2+^ concentration start in the range of 10^−4^ mol/L [65], while the highest release from Electrolyte 3 coatings with zinc is ~2 × 10^−5^ mol/L (Figure 6a). Therefore, cells will survive from a cytotoxic point of view. It should be noted that ISO 10993 requires additional tests, such as full screening for re-released ions, total organic carbon content (TOC), and organic matter content, as well as a cytotoxicity and endotoxin testing. From the results of the EDX measurement, as well as from the fabrication method and the nature of the coatings, it is evident that the TOC test results would be close to zero. Screening for organic compounds would also yield no results, since none are present. Moreover, the cytotoxicity of ZnO nano- and micro-particles on human cells has already been investigated [66]. In reference [66], the authors used a cervical cancer cell line (HeLa) and normal human fibroblasts (MSU1.1) after an incubation time of 24 h. The data obtained in this study demonstrate that both cell types exhibited a high level of viability (up to 80%) after incubation with ZnO particles up to a concentration of 10 μg/mL. A high level of toxicity (decrease in cell viability to 40%) was observed only in the ZnO concentration range of 100–1000 μg/mL [66]. Therefore, all the above-mentioned points indicate that the coatings prepared and investigated in this study should not have a cytotoxicity effect on cells.

## 4. Conclusions

In the present work, six different micro-arc oxidation coatings were prepared and investigated using different electrolyte solutions (three with and three without zinc oxide). To impart antibacterial properties to the coatings, zinc oxide powder was added to the electrolyte solutions. It has been found that the addition of the same amount of zinc oxide powder (5 g/L) to the electrolyte solutions resulted in different degrees of zinc incorporation (from (0.83 ± 0.33) at.% to (11.26 ± 0.29) at.%) into the coatings formed. However, this does not lead to a significant change in the surface morphology. The addition of zinc oxide to the electrolyte solution based on calcium oxide or hydroxyapatite leads to a decrease in the thickness of the coatings formed from (17.5 + 2.5) μm and (43.0 + 7.0) μm to (10.5 + 0.5) μm and (30.0 + 5.0) μm, respectively. Moreover, the thickness of coatings formed in the electrolyte solution based on calcium acetate monohydrate and sodium phosphate monohydrate does not change and remains (5.0 + 0.5) μm when zinc oxide powder is added. The roughness of the coating based on hydroxyapatite and orthophosphoric acid and the coating based on hydroxyapatite and orthophosphoric acid plus zinc oxide is comparable. All the coatings investigated are characterized by an amorphous-crystalline structure. The calcium phosphate and zinc oxide phases were not detectable in any of the coatings. There is a clear correlation between zinc release and the zinc content of the coatings: the more zinc is present in the coatings, the more it is released in solution. However, the relationship between antibacterial activity and zinc ion release under experimental conditions was not observed due to the different effects of zinc ions on the cell wall of Gram-positive (*Staphylococcus aureus* and Methicillin-resistant *Staphylococcus aureus*) and Gram-negative (*Pseudomonas aeruginosa*) bacteria. This effect is probably due to a threshold-related antibacterial property of zinc release, after which the effect persists at a constant level and will be an investigated in a further study. The results obtained in this study and further studies with other bacterial strains would allow the improvement of antibacterial coatings for use in surgical practice, even if the risk of microbial contamination is increased.

## Figures and Tables

**Table 1:** Elemental composition of all coatings, determined via EDX, for the elements oxygen (O), aluminum (Al), phosphorus (P), calcium (Ca), titanium (Ti), vanadium (V), and zinc (Zn), as well as the ratios of calcium to phosphorus (Ca/P) and zinc + calcium to phosphorus (Zn + Ca/P).

Sample	O (at.%)	Al (at.%)	P (at.%)	Ca (at.%)	Ti (at.%)	V (at.%)	Zn (at.%)	Ca/P	(Zn + Ca)/P
**Electrolyte 1**	56.09 ± 0.36	0.77 ± 0.02	21.56 ± 0.09	11.18 ± 0.36	9.97 ± 0.09	0.43 ± 0.05	–	0.52	–
**Electrolyte 2**	56.67 ± 0.34	1.14 ± 0.07	21.87 ± 0.09	6.66 ± 0.07	13.05 ± 0.14	0.59 ± 0.04	–	0.30	–
**Electrolyte 3**	50.38 ± 0.36	2.37 ± 0.22	5.75 ± 0.32	9.53 ± 0.31	30.51 ± 1.05	1.22 ± 0.09	–	1.62	–
**Electrolyte 1 + ZnO**	55.62 ± 0.28	0.78 ± 0.04	21.38 ± 0.15	10.75 ± 0.13	9.71 ± 0.07	0.46 ± 0.05	1.30 ± 0.09	0.50	0.56
**Electrolyte 2 + ZnO**	55.93 ± 0.49	1.09 ± 0.06	21.85 ± 0.22	6.63 ± 0.20	13.01 ± 0.26	0.65 ± 0.05	0.83 ± 0.33	0.30	0.34
**Electrolyte 3 + ZnO**	48.49 ± 0.39	1.37 ± 0.08	11.38 ± 0.17	9.78 ± 0.19	16.94 ± 0.32	0.78 ± 0.07	11.26 ± 0.29	0.86	1.90

## Data Availability

Not applicable.

## Supplementary Materials

The following supporting information can be downloaded at https://www.mdpi.com/article/10.3390/biomimetics8050444/s1, Supplementary Methods; Figure S1: Photograph of each sample surface-modified by micro-arc oxidation; Figure S2: The EDX elemental mapping micrographs of the elements oxygen (O), phosphorus (P), calcium (Ca), titanium (Ti), and zinc (Zn); Figure S3: Micrographs of water and diiodomethane droplets on the surface of each sample investigated.
