# Supplementary material for: Antibacterial Calcium Phosphate Coatings for Biomedical Applications Fabricated via Micro-Arc Oxidation
Source: Biomimetics (Basel). 2023 Sep 21;8(5):444. doi: 10.3390/biomimetics8050444 (PMC10526763; doi:10.3390/biomimetics8050444)
Supplement: Supplementary file 1 [file biomimetics-08-00444-s001.zip › biomimetics-2532417-supplementary.pdf]

Supporting Information of: Antibacterial Calcium Phosphate Coatings for Biomedical Applications Fabricated via Micro-Arc Oxidation

Anna I. Kozelskaya [1,*], Ksenia N. Verzunova [1], Igor O. Akimchenko [1], Johannes Frueh [1], Vsevolod I. Petrov [2], Galina B. Slepchenko [1], Olga V. Bakina [3], Marat I. Lerner [3], Leonid K. Brizhan [4], Denis V. Davydov [4], Artur A. Kerimov [4], Elena G. Cherempey [5], Sergey E. Krylov [6], Sven Rutkowski [1,2,*] and Sergei I. Tverdokhlebov [1,*]

[1] Weinberg Research Center, School of Nuclear Science &Engineering, Tomsk Polytechnic University, 30, Lenin Avenue, 634050 Tomsk, Russia; shumskaya_k@mail.ru (K.N.V.); akimchenko.igor@gmail.com (I.O.A.); johannes.frueh@alumni.ethz.ch (J.F.); microlab@tpu.ru (G.B.S.)
[2] Tomsk Scientific Center of the Siberian Branch of the Russian Academy of Sciences, 10/4, Akademicheskii Prospekt, 634055 Tomsk, Russia; seva-ne@mail.ru
[3] Institute of Strength Physics and Materials Science of the Siberian Branch of the Russian Academy of Sciences, 2/4, Akademicheskii Prospekt, 634055 Tomsk, Russia; ovbakina@ispms.ru (O.V.B.); lerner@ispms.ru (M.I.L.)
[4] Federal State Budgetary Institution «Main Military Clinical Hospital Named after Academician N.N. Burdenko» of the Ministry of defense of the Russian Federation», 3 Gospitalnaya Square, 105299 Moscow, Russia; brizhan.leonid@mail.ru (L.K.B.); dvdavydov@yandex.ru (D.V.D.); kerartur@ya.ru (A.A.K.)
[5] UMH LLC, 48 Govorova Street, Apt. 191, 634050 Tomsk, Russia; cherempey@mail.ru
[6] BITECA LLC, 9 Zapadnaya Street, Building 10, 143002 Odintsovo, Russia; info@biteca.ru
* Correspondence: kozelskayaai@tpu.ru (A.I.K.); rutkowski_s@tpu.ru (S.R.); tverd@tpu.ru (S.I.T.)

Supplementary Methods

Zinc Release Determination. Zinc ion concentrations were measured using the standard addition method applied to stripping voltammetry. Three different samples were prepared: a control sample, samples extracted from the coatings on substrate, and samples with known zinc concentration. All samples were measured under the same conditions. Anodic zinc peaks were simultaneously recorded as voltammetric graphs whose peak heights were proportional to the mass concentration of the element in solution. A three-electrode measuring system has been applied. For this, a voltammetric complex (STA, ITM Ltd., Tomsk, Russia) equipped with three electrochemical cells has been used, which allows simultaneously measurement of three samples. The working electrode was a mercury-film electrode on a silver substrate. A mercury film (thickness: 10 – 20 µm) was applied by wetting the silver surface with pure mercury. The reference electrode consisted of silver chloride; the auxiliary electrode was silver chloride with a surface area of 1.5 cm^2. During the measurements, the increasing rate for the potential was 100 mV/s. Oxygen was removed by UV irradiation of the background solution. For the accumulation of zinc in amalgam, the electrolytic potential was set to 1.40 V, and the time of accumulation varied from 30 s to 120 s. The signal accumulation time depends on the content of a particular element in the sample. The measurements were carried out at room temperature.

Sample preparation. To prepare the control sample 0.2 cm^3 of formic acid (reagent grade, Merck, Darmstadt, Germany) and 10 cm^3 double-distilled water were placed in a quartz beaker with a volume of 20 cm^3. The same composition was used to prepare the second sample, to which was added as a coated sample that remained in this solution for 6 hours. Finally, the third sample was identical to the second but had a known amount of zinc added. It should be mentioned that the amount of added zinc depended on the strength of the analytical signal. The calculation of the mass concentration of zinc in solution with the coated sample was carried out by comparing the voltammetric graphs of the three samples mentioned above. At least three measurements were performed for each type of coating.

Supporting Figures

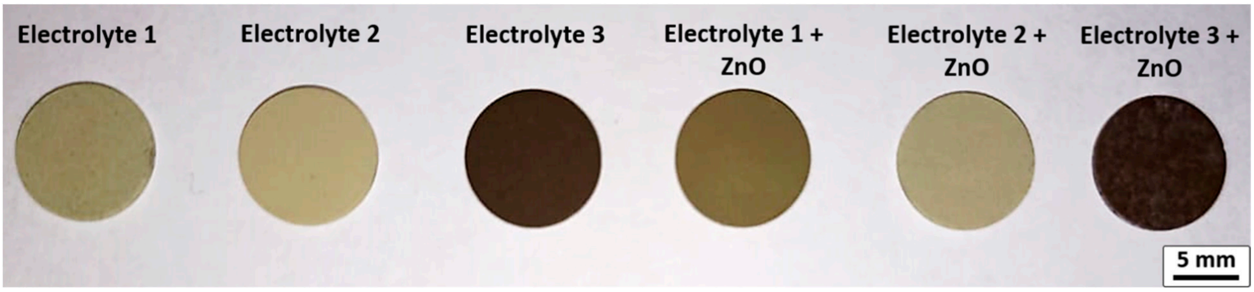

Figure S1. Photograph of each sample surface-modified by micro-arc oxidation.

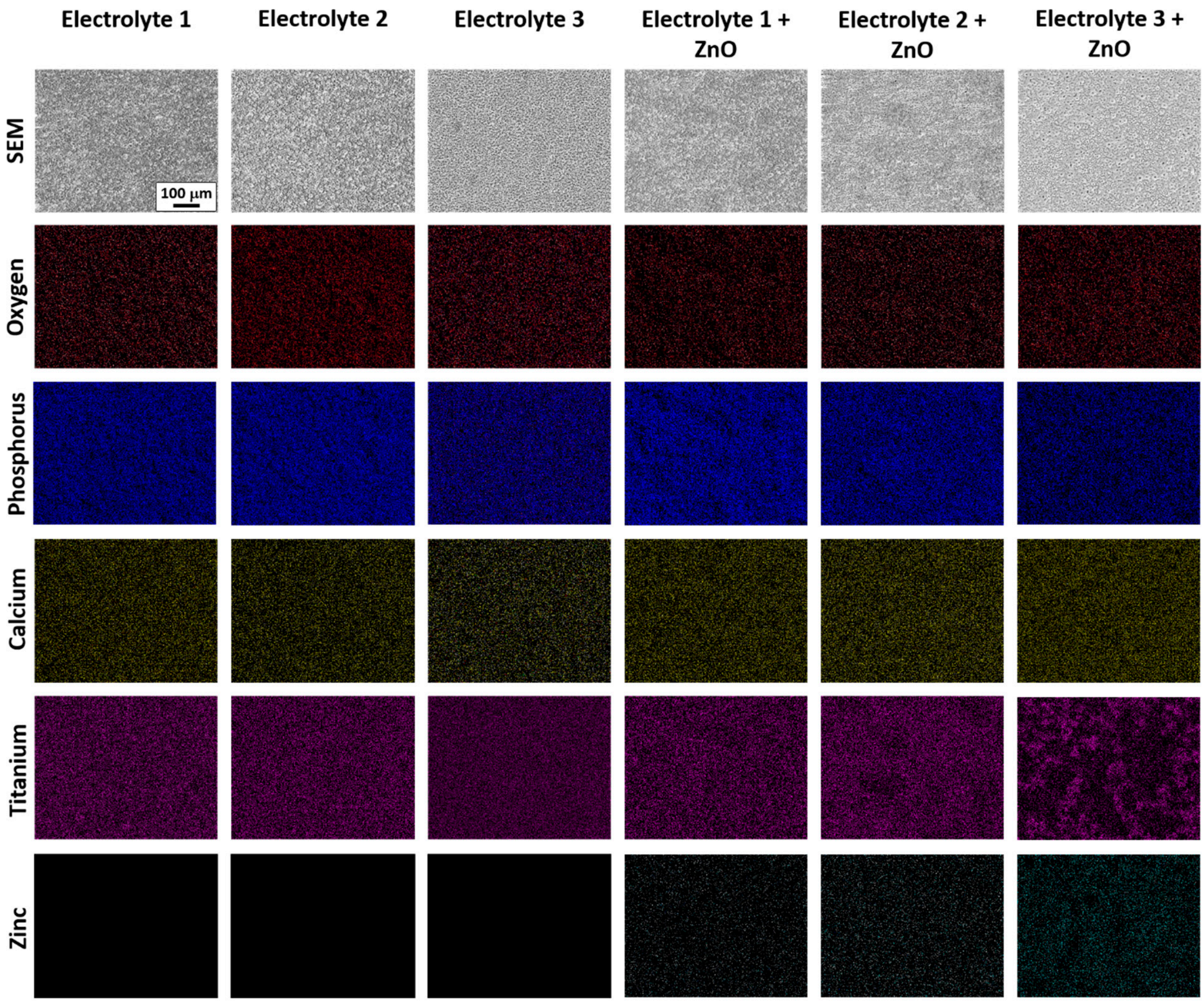

Figure S2. EDX elemental mapping micrographs of the elements oxygen (O), phosphorus (P), calcium (Ca), titanium (Ti) and zinc (Zn) for the examined coatings. The scale bar is 100 µm and representative for all micrographs shown in this figure.

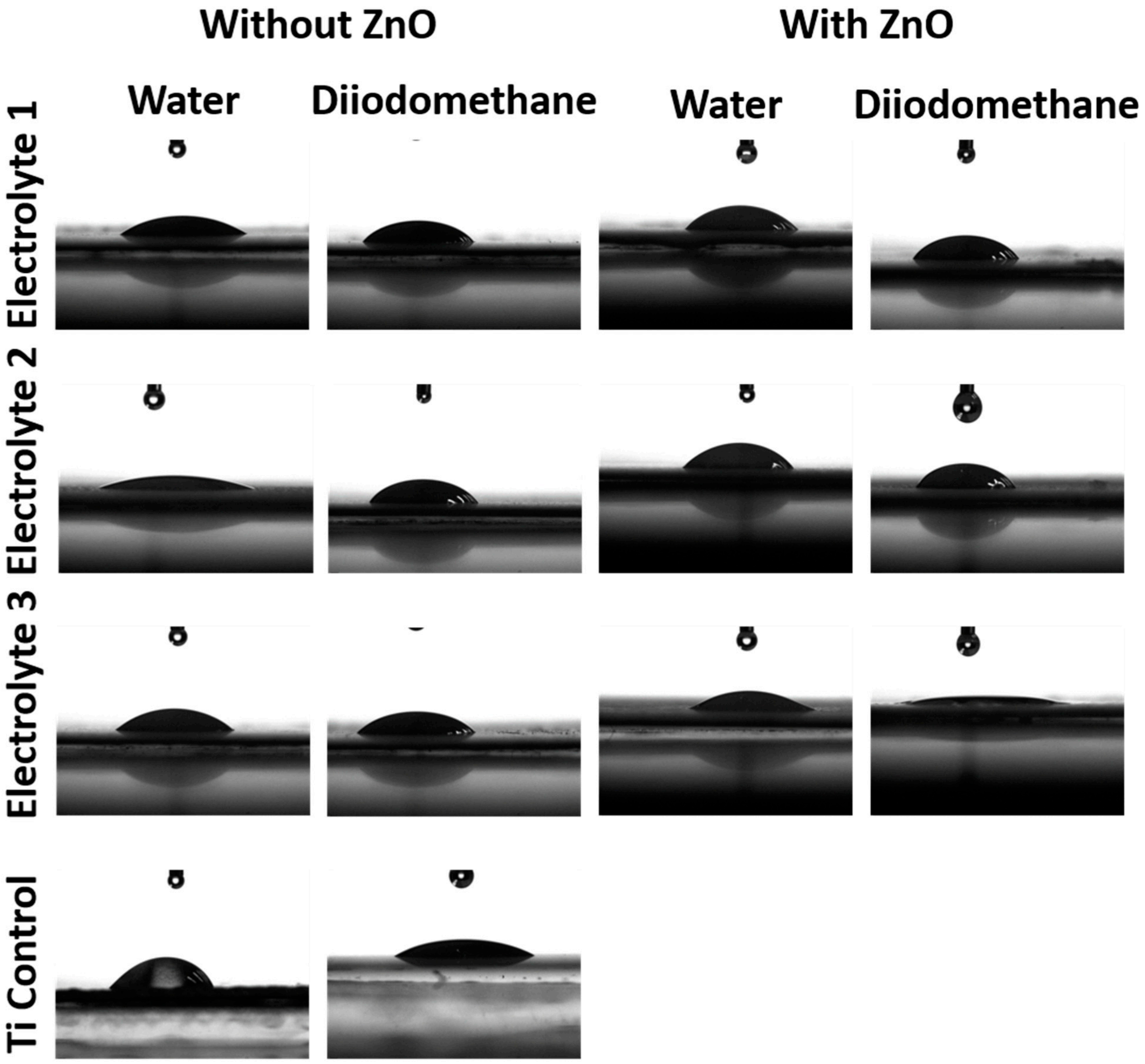

Figure S3. Micrographs of water and diiodomethane droplets on the surface of each sample investigated. The values obtained are shown in Figure 5a of the main manuscript.
